# Supplementary material for: Senescence-driven solubilization of biomass is the main source of kelp-derived dissolved organic carbon to the coastal ocean
Source: Commun Biol. 2025 Aug 7;8:1172. doi: 10.1038/s42003-025-08477-y (PMC12331991; doi:10.1038/s42003-025-08477-y)
Supplement: Supplementary file 2 — Supplementary Information [file 42003_2025_8477_MOESM2_ESM.pdf]

## Supplementary Information

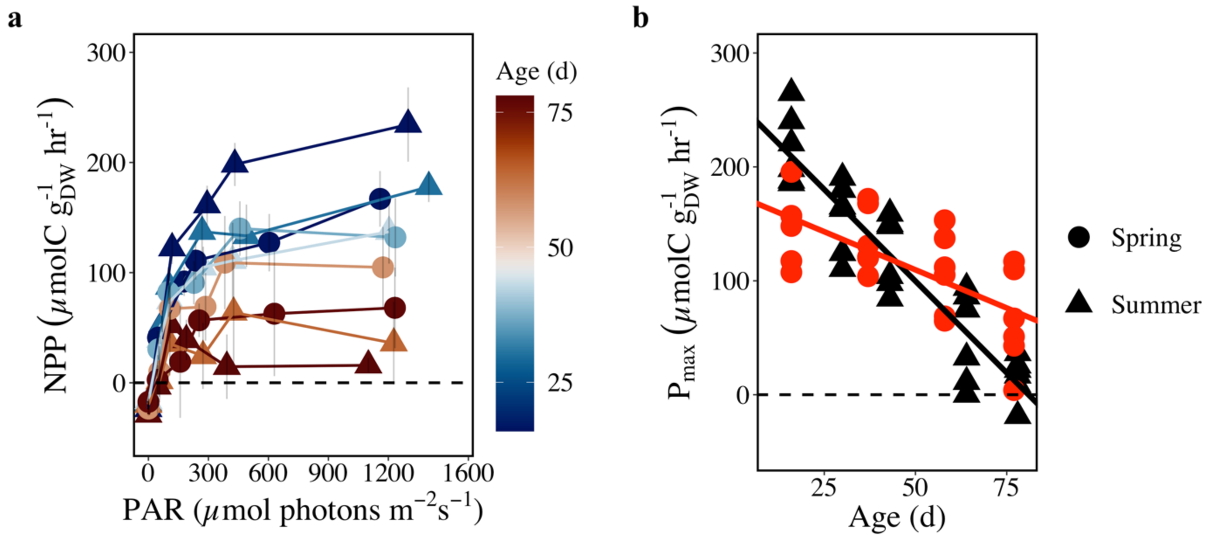


**Supplementary Figure 1. (a)** Photosynthesis-irradiance curves for giant kelp blades grouped by age and season. Error bars represent ±1SD of NPP for triplicate blades incubated at similar light levels. **(b)** Linear decrease in maximum photosynthetic rate ($P_{max}$) with age in both spring (Ordinary Least Squares Regression, $R^2 = 0.44$, $p < 0.001$, $y = -1.33x + 176.6$, $n = 24$) and summer (OLS, $R^2 = 0.85$, $p < 0.001$, $y = -3.22 + 260.7$). Each point is the photosynthetic rate of one of the six blades incubated at a saturating irradiance (> 300 μmol photons $m^{-2}$ $s^{-1}$). Red circles and solid red line are $P_{max}$ values and the Ordinary Least Squares Regression for spring, respectively. Black circles and solid black line are $P_{max}$ values and the Ordinary Least Squares Regression for summer, respectively. The dashed, horizontal line represents the transition between net respiration and net photosynthesis.

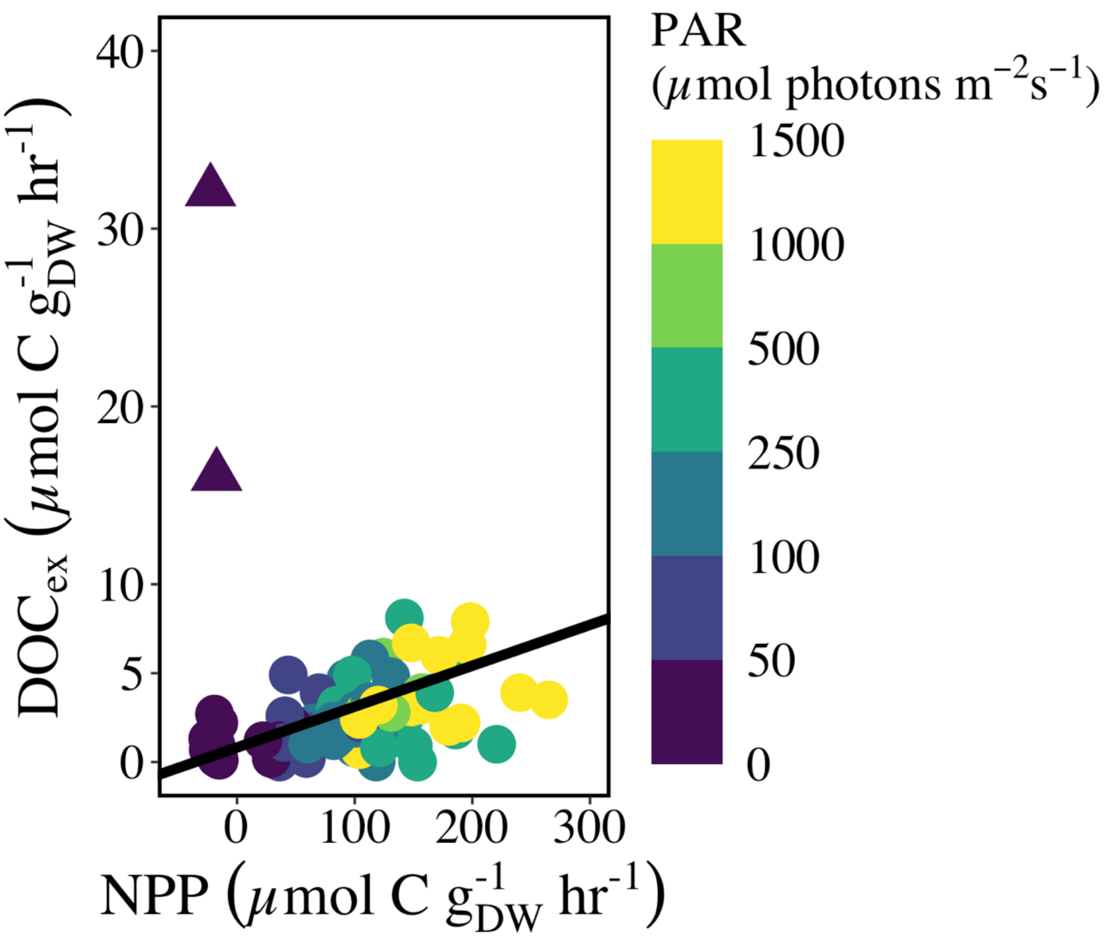


**Supplementary Figure 2**. Figure 2 in the main text including the two excluded outlier points, shown as triangles. In mature kelp incubations (age < 50 days), two blades were accidently damaged by the stir bar in the incubation chambers, resulting in elevated $DOC_{ex}$ (16.1 & 32.1 μmol C $g_{DW}^{-1}$ $hr^{-1}$) in the dark (PAR = 0). Previous to the damage, in their respective light incubations (PAR = 74 – 554 μmol photons $m^{-2}$ $s^{-1}$), these blades had $DOC_{ex}$ rates between -1.2 – 5.9 μmol C $g_{DW}^{-1}$ $hr^{-1}$ that fell along the Model II regression line shown.

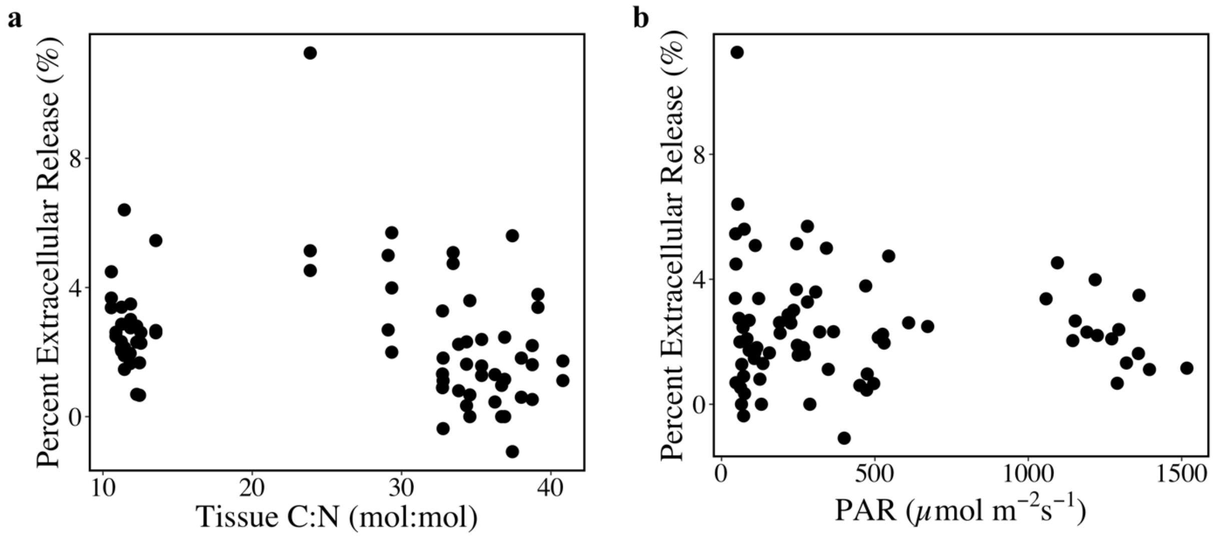


**Supplementary Figure 3. (a)** There is a weak negative, significant relationship (Model II, $p < 0.001$, $R^2 = 0.08$) between the percent extracellular release (DOC/NPP *100%) of mature kelp blades (age < 50 days) and Tissue carbon to nitrogen content (C:N), **(b)** There is no significant correlation between PER of mature kelp blades and light intensity

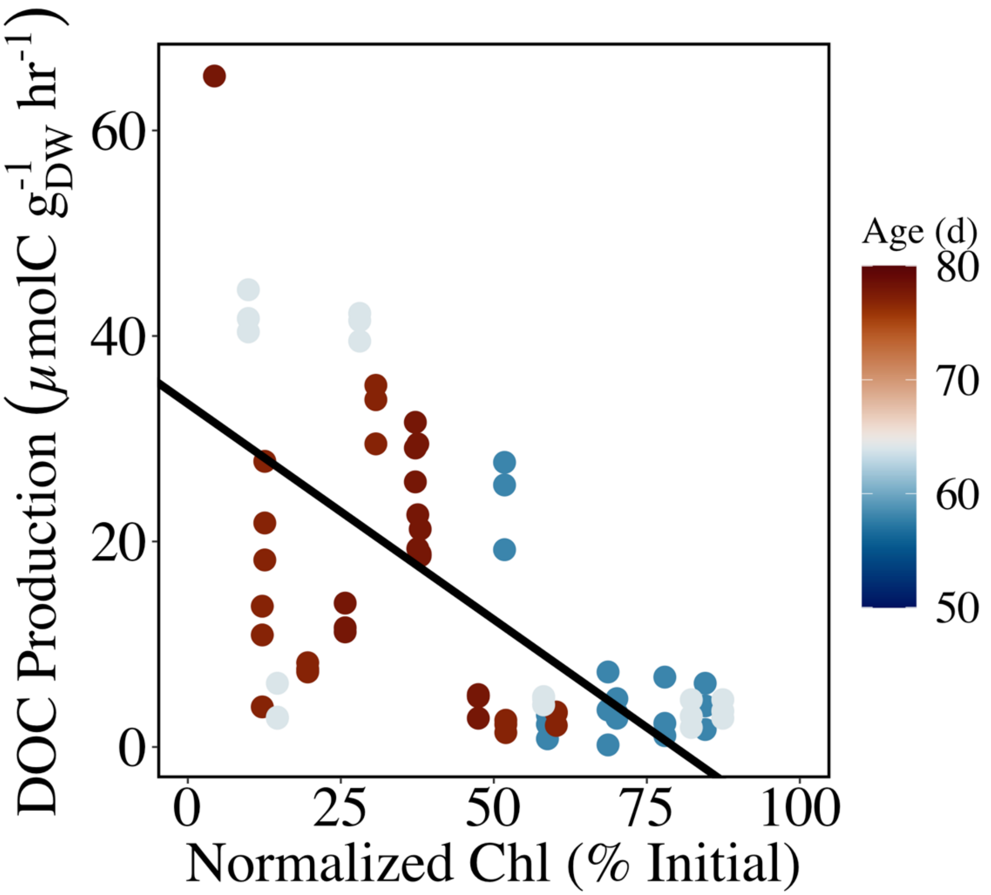


**Supplementary Figure 4.** DOC production by senescent phase kelp (>50 days of age) increases with the progressive physiological decline in kelp blade chlorophyll *a* (Chl*a*) content. Normalized Chl*a* content is the Chl*a* content of each blade (age > 50 days) expressed as a percent of the average Chl*a* content at the beginning of each cohort sampling (age = 16 days). Solid line is the significant model II regression result (y = -0.42x + 33.4, $R^2$ = 0.35, p = 4.1e$^{-08}$).

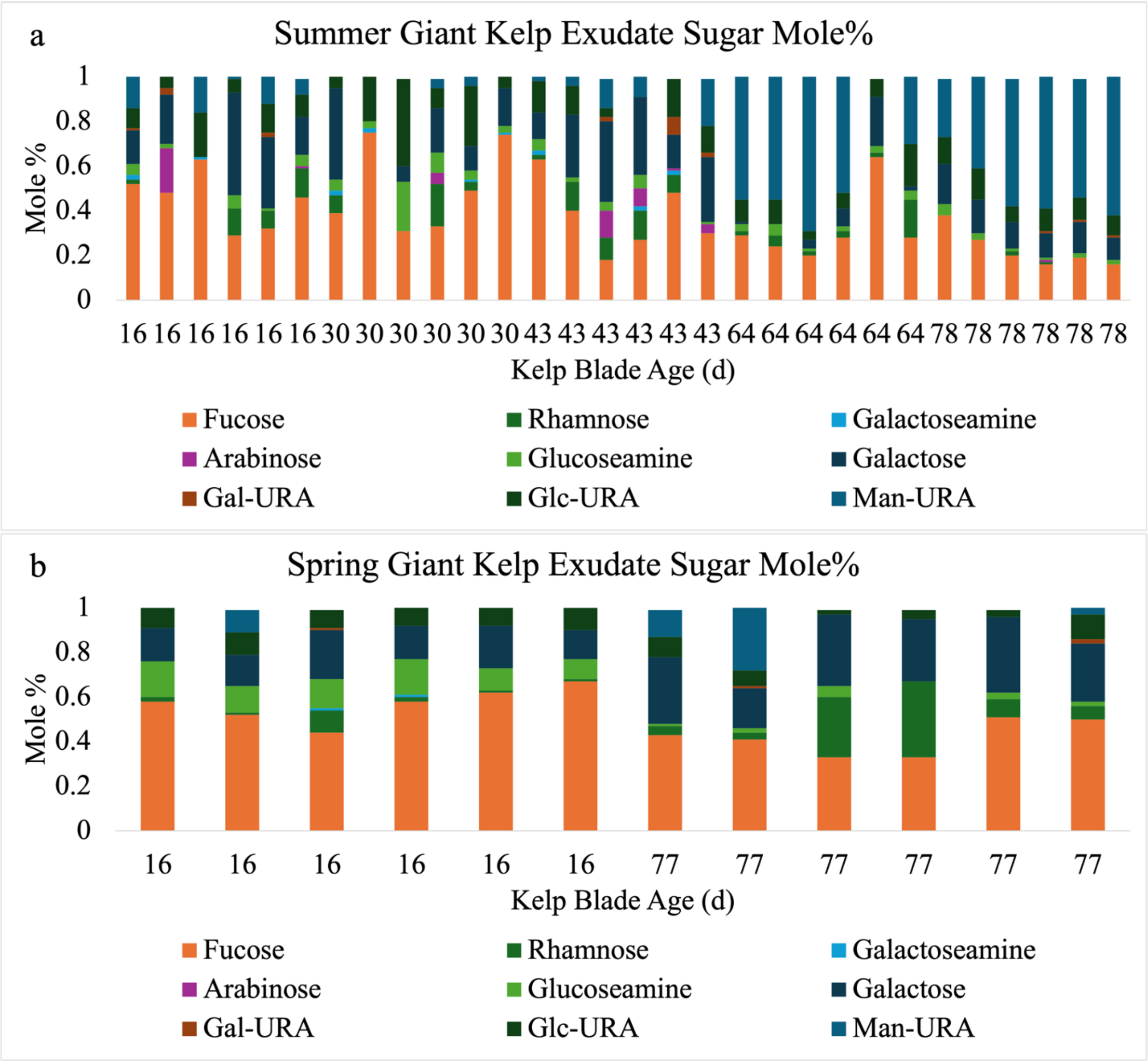


**Supplementary Figure 5.** The relative contribution (mole%) of individual sugar monomers to the total sugar monomers released by giant kelp at different tissue ages in the **(a)** Summer and **(b)** Spring. Abbreviations: Glc-URA (glucuronic acid), Gal-URA (galacturonic acid), Man-URA (mannuronic acid). Repeat ages on the x-axis are values from replicate blades incubated at each tissue-age step.

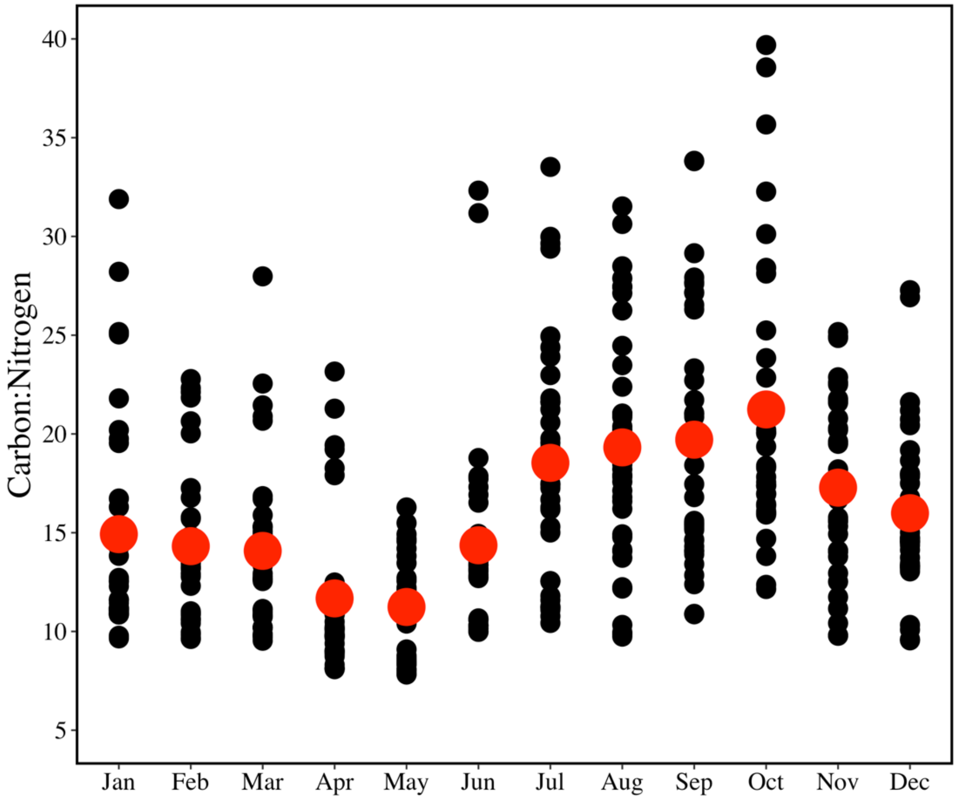


**Supplementary Figure 6.** Monthly giant kelp tissue carbon to nitrogen (grams: grams of kelp dry weight) sampled from Mohawk Reef between the years 2002 – 2021 (black circles). The large red dots are the monthly means over the time series record. Data was retrieved from the SBC-Long Term Ecological Research project (sbclter.msi.ucsb.edu/data/).

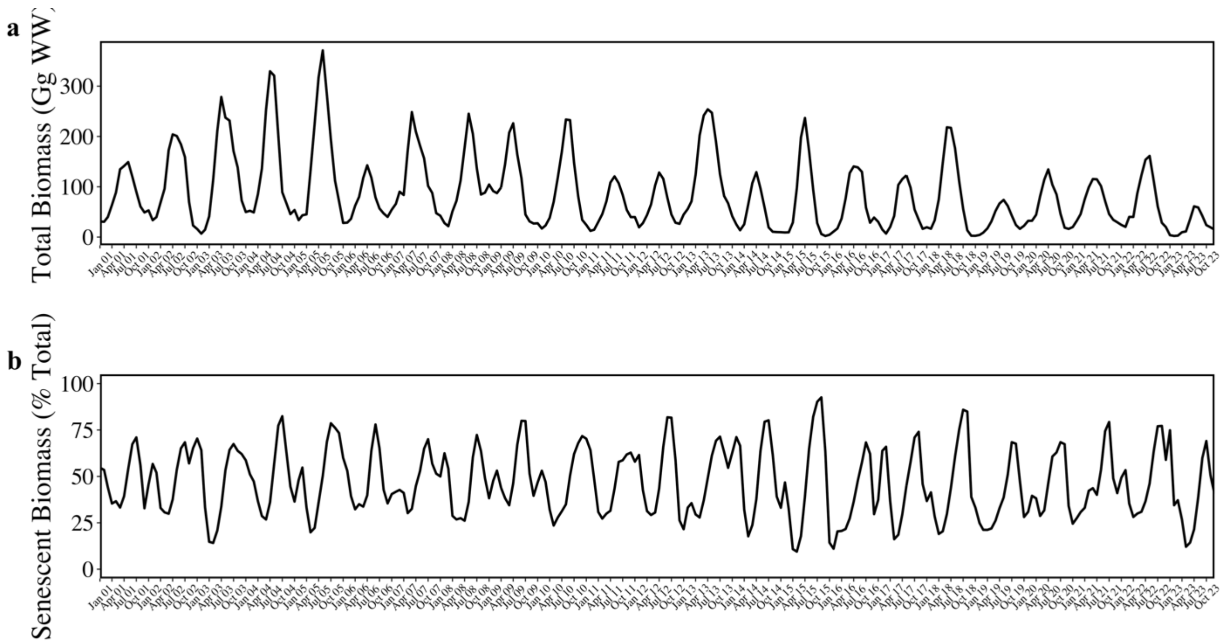


**Supplementary Figure 7:** Data presented in Figure 4 as a continuous series. Intra- and interannual variability in giant kelp canopy biomass (in Gg of wet weight) and physiological state estimated from Landsat imagery across the central and southern California region. **(a)** Monthly estimates of giant kelp canopy biomass between 2001-2023 derived from Landsat 7, 8, and 9 multispectral sensors. Note: 1 Gg = 1000 metric tons. **(b)** Percentage of total monthly biomass in panel A that is senescent (> 50 days old).

**Supplementary Data 1:** (attached as a separate excel file). Sheet 1: Values for all data generated from this study and used to generate figures. Sheet 2: Description of data variables.

**Supplementary Table 1**. Summary statistics of the nine separate sampling and incubation events for the spring and summer cohorts including the date kelp was sampled for each incubation. Included are the mean ± 1 standard deviation of blade physiological measurements for the six replicate blades incubated at each time and the range of incubation light levels, rates of net primary production and net DOC exudation for the 18 rate measurements made per sampling event.

| **Season** | **Date** | **Age (d)** | **Blade C:N (mol:mol)** | **Blade Chl:C (mg $g^{-1}$)** | **PAR (µmol photons $m^{-2}$ $s^{-2}$)** | **NPP (µmol C $g_{DW}^{-1}$ $hr^{-1}$)** | **$DOC_{ex}$ (µmol C $g_{DW}^{-1}$ $hr^{-1}$)** |
|---|---|---|---|---|---|---|---|
| Summer | 8/9/23 | 16 | 35.0 ± 3.9 | 6.8 ± 1.7 | 0 - 1359 | -24.6 – 264.9 | -0.5 – 8.1 |
| Summer | 8/23/23 | 30 | 35.0 ± 1.9 | 6.4 ± 0.4 | 0 - 1517 | -18.5 – 190.5 | -1.2 – 5.9 |
| Summer | 9/5/23 | 43 | 33.9 ± 6.2 | 7.6 ± 2.2 | 0 - 1290 | -19.2 – 159.0 | 0.0 - 6.7 |
| Summer | 9/26/23 | 64 | 40.4 ± 3.3 | 3.3 ± 2.1 | 0 - 1325 | -23.4 – 93.9 | 1.9 - 44.5 |
| Summer | 10/10/23 | 78 | 42.0 ± 2.1 | 3.0 ± 0.9 | 0 - 1140 | -30.3 – 61.0 | 2.8 - 65.3 |
| Spring | 4/17/24 | 16 | 11.4 ± 0.7 | 13.5 ± 4.4 | 0 - 1272 | -23.7 – 195.6 | 0.2 - 6.6 |
| Spring | 5/7/24 | 37 | 12.1 ± 0.8 | 14.8 ± 3.4 | 0 - 1361 | -25.0 – 172.0 | 0.2 – 6.0 |
| Spring | 5/28/24 | 58 | 11.5 ± 0.8 | 9.7 ± 2.8 | 0 - 1261 | -28.8 – 153.2 | 0.2 - 27.7 |
| Spring | 6/16/24 | 77 | 17.8 ± 4.0 | 5.0 ± 2.5 | 0 - 1331 | -28.0 – 117.0 | 1.4 - 35.2 |
