# Supplementary material for: Senescence-driven solubilization of biomass is the main source of kelp-derived dissolved organic carbon to the coastal ocean
Source: Commun Biol. 2025 Aug 7;8:1172. doi: 10.1038/s42003-025-08477-y (PMC12331991; doi:10.1038/s42003-025-08477-y)
Supplement: Supplementary file 3 — Description of Additional Supplementary Files [file 42003_2025_8477_MOESM3_ESM.docx]

Description of Additional Supplementary Files

**File name:** Supplementary Data 1

**Description:** The source and meta-data behind the incubation conditions and graphs in the paper.
